# Cell jamming transitions can affect regulatory protein gradients and prime evolutionary divergence

**DOI:** 10.1098/rsif.2025.0186

**Published:** 2025-11-12

**Authors:** Alexander V. Badyaev, Cody A. Lee, Maxwell J. Gleason, Georgy A. Semenov, Sarah E. Britton, Carmen Sánchez Moreno, Renée A. Duckworth

**Affiliations:** ^1^ Department of Ecology and Evolutionary Biology, University of Arizona, Tucson, AZ, USA; ^2^ College of Human Medicine, Michigan State University, East Lansing, MI, USA; ^3^ Department of Ecology and Evolutionary Biology, University of Colorado, Boulder, CO, USA; ^4^ Department of Ecology, Behavior and Evolution, University of California San Diego, La Jolla, CA, USA; ^5^ Department of Molecular and Cellular Biology, University of Arizona, Tucson, AZ, USA

**Keywords:** cell morphology, rigidity transition, criticality, developmental compartmentalization, protein networks, morphogen gradients, population divergence

## Abstract

A long-standing goal of evolutionary developmental biology is to identify the rules by which processes governing individual cells scale up to organism-level patterning. The viscoelastic properties of embryonic tissues imply collective cell behaviours, leading to the expectation that gene regulatory networks should capitalize on the material properties of tissues. Here, we show that large-scale variation in morphogenesis can be traced to cell-level dynamic. In avian beak primordia, we find that fields of mesenchymal cells undergo cycles of local jamming that predictably change coordination of cell shapes and movements. These cycles, in turn, alter the spatial reach of regulatory proteins, shaping their gradients in relation to tissue mechanical state. Long-range gradients of proteins most sensitive to local jamming differ the most across populations and, through their priming of tissue compartmentalization, can facilitate evolutionary divergence in beak morphology. Jamming transitions might thus allow these tissues to reconcile seemingly contradictory needs: robust maintenance, facilitated by jamming phase that resets or synchronizes cells, and adaptive flexibility, promoted by unjamming phase, that allow rearrangements, explorations or expansions. These transitions can also integrate stochastic physical processes and biological regulation allowing local rules governing cell behaviours to propagate to tissue-level patterning, ultimately promoting diversification and plasticity while preserving robustness.

## 1. Introduction

Morphogenesis of multicellular organisms occurs at the level of tissues but is produced by coordination among thousands of individual cells, requiring integration of multiple processes and consistency of rules across vast spatial and temporal scales. Mechanisms at the interface of physical and biological processes are well suited for this integration: genetic and biochemical controls routinely capitalize on predictable material properties of cells and tissues to produce scale expansion, pattern formation, time measurement and compartmentalization [1–4].

A prime example of this integration is the transient association of mechanistic and regulatory processes in the jamming transition of cell groups during tissue reorganization [5–9]. During early embryogenesis, cells in homogeneous fields routinely undergo spontaneous rearrangements caused by the accumulation and dissipation of the compressive stress that cells impose on each other. This process leads to the transient appearance of two tissue states: a glassy-like jammed state of slow-moving cells and a fluid-like unjammed state of fast-moving cells. Although the bidirectional transition between jammed and unjammed states is ubiquitous in development, wound repair and cancer [9–13], how regulatory and cellular processes interact during these transitions and their evolutionary consequences are poorly understood.

In developing tissues, anything that affects cell shape, density, growth, competition, migration, adhesion or differentiation can trigger jamming transitions [7,14,15]. Furthermore, in biological systems, unlike their physical counterparts, jamming transitions are often both active and passive: they can be induced by cells themselves through changes in shape, cycle synchronization or adhesion propensity, or can be imposed on cells by surrounding tissues of different rigidity or by crowding in a confined space [16–18]. In all of these cases, however, a local process affecting just a few cells leads to larger-scale structural reorganization of the tissue without additional developmental controls at the tissue level [1,14,18]. These processes are thus well placed to underlie criticality of developmental systems [19]. This is because they provide links among levels and components of disparate organization and nature (e.g. physical versus biological) in such a way that the regulatory system at each level stabilizes a large-scale expansion arising through incremental variation at the preceding stage but does not cause these transformations directly. The search for such processes is a long-standing goal of evolutionary developmental biology [20,21].

The multitude of potential causes of jamming transitions precludes its monopolization by any one biological regulator during evolution. Instead, regulatory networks are expected to capitalize on the effects of these transitions [22–25]. Furthermore, cell mechanical state and the synthesis of regulatory proteins are directly linked [26,27], both through the effects of jamming transitions on membrane-to-nucleus distances, cytoplasmic and nuclear biochemistry, and accessibility of cell and nuclear membranes (as a function of cell isomorphy and membrane porosity [4,28,29]) and through the effects of tissue material properties on morphogen propagation and concentration [28,30,31]. Coupled with the effects of cell jamming on the scale of cell coordination, this integration should result in mesoscale consequences for developmental compartmentalization that should be detectable in evolutionary diversifications.

**Figure 1.**
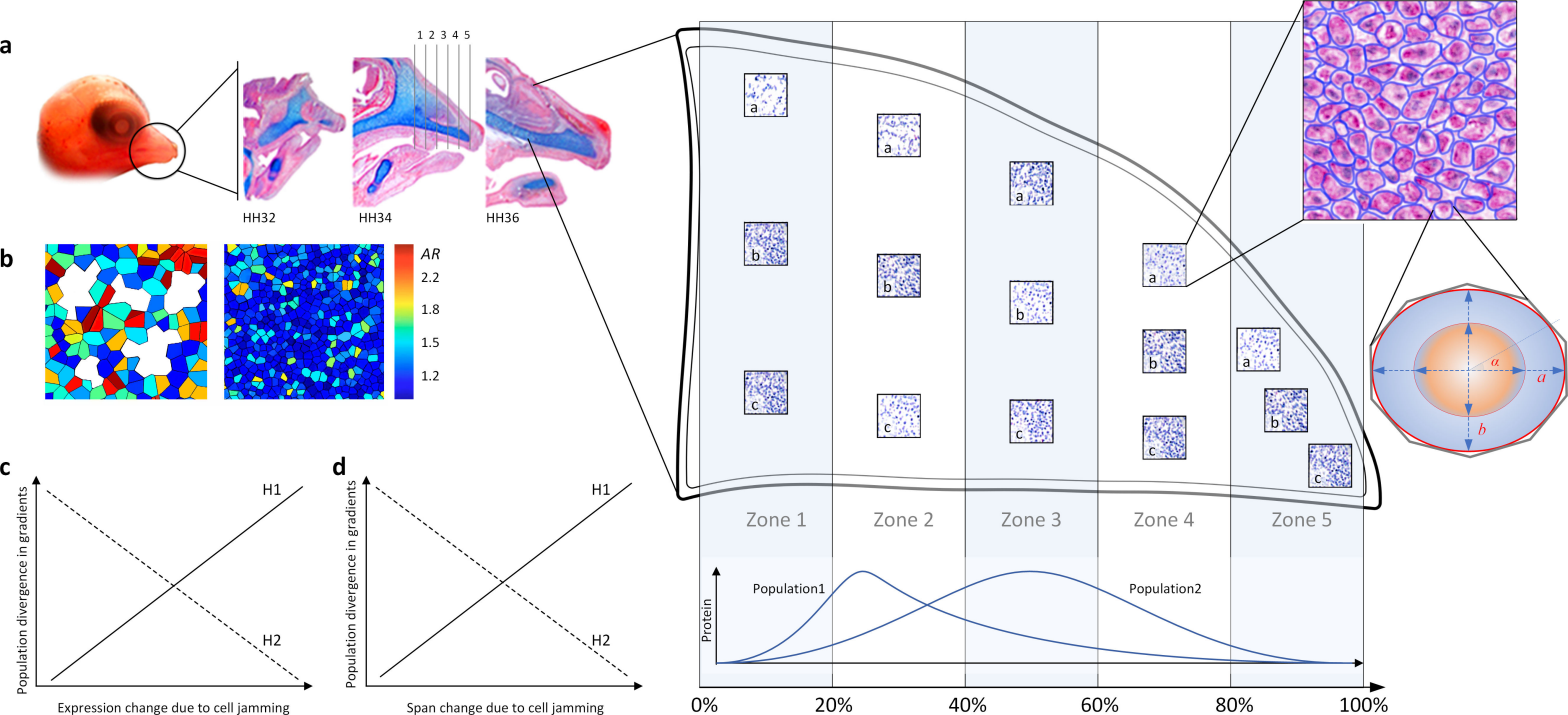
Schematics of sampling protocol and predictions. (a) Areas of interest were extracted from each beak midline section throughout developmental stages (three stages are shown as examples) and partitioned into five equal 20% zones along anterior–posterior (AP) axis. Three fields of views (a, b, c, each 71 × 71 µm) were selected within each zone, and all cells were measured within each field of view. Cell and nucleus shape were approximated with a customized algorithm, and the major and minor axes (*a*, *b*), coordinate of each cell axes and centroids, alignment angle (*α*), perimeter, and area were calculated for the best fitting ellipse. Aspect ratio (AR)=a/b and average cell shape index $\overset{-}{p}$=$Perimeter/\sqrt{Area}$ were calculated for each cell, and cell jamming was categorized for each field of view. Populations can differ in AP gradient in protein expression. (b) Schematics of AR distribution in heatmap gradient in unjammed (left) and jammed (right) fields of view. (c and d) Cell-jamming associated protein expression can delineate (H1) or counteract (H2) tissue compartmentalization. (c) Mechanosensing proteins can facilitate tissue compartmentalization by delineating directions of population divergence in protein gradients through local effects (such as changes in protein connectivity). (d) Jamming-associated changes in protein expression can affect AP protein gradients, priming population divergence in these gradients. Alternatively (H2), the mechanosensing proteins can counteract tissue compartmentalization associated with jamming either (c) locally, by accommodating stochastic variation in protein expression caused by jamming or (d) globally, by equalizing spatial reach of proteins across tissues.

Here, we show that this is indeed the case for ontogeny of beaks across recently established house finch (*Haemorhous mexicanus*) populations [32]. We first document the ubiquity of local cell jamming transitions in homogeneous fields of neural crest mesenchyme (NCM) of beak primordia. We then show that the propagation of proteins most consistently expressed throughout avian beak development [33–36] is linked to these jamming cycles. Next, we examine population divergence in proteins whose local reach is altered by cell jamming cycles (figure 1). We first show that the cell jamming transitions are ubiquitous across developmental contexts and reflect an interdependence of cell shape and its variability. We then discuss how this consistent phenomenon can nevertheless lead to population‐level branching of outcomes seen in divergent long-range gradients of regulatory proteins. We also discuss whether the scale-expansion effects of cell jamming can be an origin of scaling transformations of avian beak morphogenesis [37].

## 2. Methods

### 2.1. Data collection and sample sizes

Twenty-two study populations of the house finch were grouped into five geographic groups based on their genetic similarity (electronic supplementary material, table S1; [38]). Freshly laid eggs were numbered and either allowed to develop in nests with direct monitoring of incubation onset [39] or placed in portable incubators programmed to house finch incubation parameters [40]. When embryos reached the required developmental stage, eggs were opened, and embryos were placed in a Petri dish with phosphate-buffered saline (PBS) and photographed under a Series 80 microscope at ×10 magnification. Embryological stage was confirmed [41] and embryos were stored in PAXgene Tissue System (PreAnalytics) that allows simultaneous storage of RNA, DNA and tissues in the field [42–44]. Upon return to the laboratory, beaks were separated, cryosectioned at 8 µm (approx. one cell thickness) and stored at −80°C. Thirteen sections per individual were obtained at beak midline: one section was stained with Alcian blue haematoxylin and eosin (H&E; U. Rochester MC) to delineate the histological and anatomical area of interest (AOI); 12 were used in immunohistochemical (IHC) analysis of proteins described below. Sections were photographed with a DP74 camera using an Olympus BX43F microscope under ×4, ×20 and ×40 magnifications. To increase statistical power for some tests, developmental stages were combined into three groups: HH 25–31, HH 32–33 and HH 34–36 (electronic supplementary material, table S1).

### 2.2. Cell measurements and method validation

We measured shape, density and alignment of 454 988 NCM cells in 7408 ×20 fields of view (60–300 cells in each, hereafter cell groups) distributed across five zones of the upper beak of 352 embryos (figure 1a, electronic supplementary material, table S1, [45,46]). Because our focus was the behaviour of individual cells, we only sampled cells either prior to formation of cell condensations or outside of forming condensations and differentiating tissues. We also excluded cells that were overlapping, dividing or damaged during sectioning. Cell measurements and IHC expression were conducted within an AOI of the upper beak that was delineated based on landmarks homologous across developmental stages and confirmed with H&E histological staining [47]. Briefly, the upper beak AOI was from the point of inflection of the upper beak, to the lower outer tissue of the brain/eye to the inner edge of the mouth, just past the palatine process, but not including the palatine process, to the tip of the beak, along the inner edge of the egg tooth and back to the point of upper beak inflection.

Each AOI was divided into five equal zones (figure 1a) based on AOI coordinates and using an overlying scalable grid [47]. Three non-overlapping fields of view of 71 × 71 μm (a, b, c) were randomly selected within each zone and imaged under ×40. Only NCM cell fields were sampled, and care was taken to avoid tissue gaps and folds, epithelial boundaries and capillaries. The photographs were exported into ImageJ [48], retaining their calibration and converted to black and white. A custom script [47] was used to subtract the image from its background, enhance boundaries and to apply an intensity threshold.

We validated our methods in two ways. First, we manually measured cells and nuclei to assess the accuracy of an automated workflow [47] and to verify that nuclei size was a good proxy of cell size. We selected 50 individuals with membranal β-catenin expression (IHC methods, below) across nine developmental stages and imaged the three replica fields of view mentioned above under ×40 magnification. Between 10 and 30 cells were sampled from each replica. Photographs were imported into ImageJ, and the polygon selection tool was used to manually trace the nucleus and measure area (μm^2^), centroid coordinates, perimeter (μm), major and minor axes of best fitting ellipse, angle of major axis, circularity and Feret length (the longest distance between any two points in the nucleus; figure 1a). The same measurements were then repeated for the entire cell. A total of *n* = 1020 cells were manually measured for this validation. High and repeatable correlations between cell and nucleus area (electronic supplementary material, figure S12) and shape measures (min Feret is shown, electronic supplementary material, figure S13) confirmed that nucleus size can be used as a proxy measurement of cell size [49–51] and that deformation of the mechanosensitive nucleus is proportional and causally linked to the cell jamming transition [52–56]. This enabled us to extend cell morphology measures to IHC assays with nuclear counterstaining. Second, in the automated workflow [47], three replicas were obtained in each of the five zones as above; the resulting 71 × 71 μm fields of view were photographed under ×20 and exported to ImageJ where a custom-script-recorded nucleus area (μm^2^), centroid coordinates, perimeter (μm), major and minor axis lengths of the best fitting ellipse, aspect ratio and Feret length, angle and coordinates (figure 1a).

### 2.3. Inferring cell jamming state from cell measurements

Variation in cell shape in cell aggregation determines the distribution of contact lengths among neighbouring cells and is a sufficient predictor of the jamming transition in groups where all cells experience similar internal and external forces [57], such as in cell monolayers [10,11]. However, in most three-dimensional developing tissues that undergo rapid expansion with collective cell movements and rearrangements, such as the NCM cells studied here, neighbouring cell groups can differ in fluidity [13,25] and thus exert and experience variable forces from other cell groups. In these cases, additional cell metrics, such as cell density and cell alignment [58], might be required to infer the onset of jamming. However, even in three-dimensional cell systems, where other cells are treated as a medium (instead of a substrate as in monolayers), the relationship between cell shape and the onset of jamming is still fundamentally related to critical values of the length of contacts and contact distribution among deformable cells [59] and corresponding changes in accumulation of compression stress [60]. The main complication for inferring cell mechanical state from geometry alone in such systems comes not from geometry, but from a cell’s ability to actively change its shape, elasticity and other behaviours, and thus direct their own jamming and unjamming transitions. Furthermore, even in purely physical systems, critical shape values associated with jamming depend not only on dimensionality of the system but also on context. For example, ellipsoid particles have higher thresholds to the jamming transition than round particles, yet easier alignment of ellipsoid particles brings them closer to the jamming transition [61]. Broadly, however, the same principles apply as indicated by (i) universality of the relationship between shape and its variability in jamming transitions and (ii) broad similarity in properties of jamming transitions in physical and biological systems and in monolayers and three-dimensional tissues [59]. Furthermore, metrics utilized by the studies of jamming in three-dimensional tissues, such as critical values of the index of asphericity (*p*^2^/4π*a*) [59] are geometrically related to the critical values of the cell shape index (*p*) that we used here in our analysis of cells on two-dimensional slides. Thus, our combination of measures can be used to infer jammed and unjammed states of cell groups during development.

### 2.4. Classification of cell jamming/unjamming

We classified cell jamming for each cell group (a, b or c; figure 1a) by applying a combination of five indicators of cell jamming [15,16,57–59] and calculating agreement among them. First, we calculated the cell shape index $\bar{p}$=$\frac{Perimeter}{\sqrt{Area}}$ and used p* = 3.81 [58,59] as a critical value below which cells are jammed, such that any cell rearrangements require cell shape changes [10] (minimum value of *p* is 3.54 at which a cell is a circle). Second, to specifically examine the effect of shape elongation, we calculated cell aspect ratio (*AR*)=$\frac{a}{b}$ and its variability (standard deviation (s.d.)) and used s.d. (*AR*) = 0.5 (corresponding to *AR* ≈ 1.6 in our sample, see below) as a critical value below which cells are increasingly uniform and round and are jammed [11]. Third, we calculated the s.d. of cell polarization in cell groups (angle *α* in figure 1a) and used s.d. (*α*) = 0.5 as a critical value below which cells within a group are aligned [58]. Fourth, we calculated the total area occupied by cells within the 71 × 71 μm field of view and used the critical value of 95% as a cut-off above which cells are jammed. Fifth, we calculated *AR* for all cells within a group and calculated the ratio of cells with *AR* < 1.6 to the number of all cells within a group and used the critical value of 70% above which a cell group was considered ‘jammed’ [59]. We then compared agreement scores (Cohen’s kappa) across all trios, quartets and quintets of these metrics. We found that the combination of cell shape index (*p*), variation in cell alignment (s.d. (*α*)) and variation in aspect ratio (s.d. (*AR*)) showed the highest agreement among all the metric combinations (Cohen’s κ (overall) = 0.46 (95% CLI: 0.39-0.53), McNemar’s S = 123.05, *p* < 0.001). Agreement on ‘jammed’ classification among these three indices was higher than the agreement on ‘unjammed’ classification (Cohen’s κ = 0.53 (0.48–0.58) versus Cohen’s κ = 0.39 (0.34–0.44)). The reliability of these criteria for repeatable categorization of NCM cell jamming was also confirmed by a larger companion study [62].

### 2.5. Protein selection and immunohistochemistry

We measured the expression of eight evolutionarily conserved proteins that play a central role in avian beak development [33,35,62–70]: β-catenin, bone morphogenic protein 4 (Bmp4), calmodulin 1 (Calm1, CaM hereafter), dickkopf homologue 3 (Dkk3), fibroblast growth factor 8 (Fgf8), Indian hedgehog (Ihh), transforming growth factor beta 2 (TGFβ2) and wingless type 4 (Wnt4). These regulatory proteins include signalling ligands (TGFβ2, Wnt4, Bmp4, Fgf8, Dkk3, Ihh), a calcium-binding messenger protein (CaM) and a key intracellular signal transducer and transcriptional co-activator (β-catenin). Together, they function in major developmental pathways such as TGF-β, Wnt, FGF, Hedgehog and calcium signalling.

Sections were blocked with an endogenous peroxidase (2% H_2_O_2_) for 20 min, washed in tris-buffered saline (TBS), blocked with 2% bovine serum albumin plus 5% normal goat serum in TBS for 1 hour. Avidin-Biotin blocking kit (Abcam) was used per manufacturer’s instructions before applying antibodies. Samples were incubated for 18 hours with a primary antibody at 4°C, washed in TBS, incubated at room temperature for 1 hour with a secondary antibody and washed again with TBS. Appropriate dilution of the primary antibody was determined by running preliminary trials with serial dilutions. Primary antibodies included anti-β-catenin (610153, 1 : 16 000, BD Transduction Laboratories), anti-CaM (sc-137079, 1 : 15, Santa Cruz Biotechnology), anti-Wnt4 (ab91226, 1 : 800; Abcam), anti-TGFβ2 (ab36495, 1 : 800, Abcam), anti-Bmp4 (ab118867, 1 : 100, Abcam), anti-Ihh (ab184624, 1 : 100, Abcam), anti-Dkk3 (ab214360, 1 : 100, Abcam) and anti-FGF8 (89550, 1 : 50, Abcam). Three different secondary antibodies (Biotinylated Goat Anti-Mouse IgG, BP-9200; Biotinylated Goat Anti-Rabbit IgG, BP-9100; Biotinylated Goat Anti-Rat IgG, all 1 : 200, Vector Labs) were used depending on the host primary antibodies. No primary controls were incubated with TBS instead of primary antibodies. During the optimization of assays, controls were also run with just the secondary antibody to verify there was no non-specific binding. Validation assays, including isotype controls for specificity of stains, are shown in electronic supplementary material, figures S2–S10. Reactions were visualized with either diaminobenzidine (DAB, Elite ABC HRP Kit, PK-6100, Vector Labs) or Vector Red Alkaline Phosphatase substrate and Vectastain ABC‐AP Kit (AK-5000, Vector Labs). Haematoxylin was used to counterstain nuclei. Each slide contained four sections, and the three slides (12 sections per embryo) were run with the following grouped antibodies: (i) β-catenin, Fgf8, Tgfβ2 and no primary control, (ii) Bmp4, Wnt4, Ihh and no primary control, and (iii) Dkk3 and no primary control, and CaM and no primary control. Suitable upper beak midline sections with expressed proteins (*n* = 5044; [45]) were imaged and named according to embryo ID, protein, developmental stage and IHC run to enable automated processing as described in [47]. Our processing protocol randomized assignment of sections from different populations and stages to IHC runs [45].

### 2.6. Quantification of expression and spatial reach of proteins

We used a custom script to measure protein expression across AOIs [47]. Briefly, the script partitioned AOIs into five equal zones (20% of the anterior–posterior span), converted the image to black and white, applied a specific threshold for each protein expression and outputted the area of expressed, unexpressed and total tissues for each zone and for the entire AOI. Spatial reach was a percentage of anterior–posterior span of five zones (in 20% increments) over which the expression of protein was significantly (*p* ≤ 0.05) correlated (after [22]). Only adjacent zones were included in calculations. Zones were considered ‘unjammed’ if at least two of the three cell groups (a, b, c) were in the unjammed state, and ‘jammed’ if at least two cell groups were in the jammed state (figure 1a). All zones across spatial reach had to be in the same state to be included in calculations. Electronic supplementary material, figure S11 shows details and examples of calculations.

### 2.7. Statistical analyses

To achieve normal distribution, reduce skewness and stabilize variance, we used the Box-Cox transformation with λ = 0.5 for raw data on protein expression, log10 transformation for cell morphology measures and arcsine transformation for cell density proportional measures. We used a mixed-effects model with restricted maximum likelihood to examine the effects of cell jamming state on protein expression. Population and embryo ID were included as random effects. Divergence in anterior–posterior distribution of proteins among embryos was examined as a statistical interaction between Zone and embryo ID. Population divergence in anterior–posterior protein gradients was modelled as a statistical interaction between Population and Zone—i.e. population-specific patterns of protein expression in each zone. We computed least-squared means of protein expression for each cell state (jammed and unjammed) and assessed the significance of these means and the difference between them with a Sidak adjustment for multiple comparisons (electronic supplementary material, table S4). To directly evaluate the contribution of both random and fixed effects to variance in protein expression explained by mixed effects models, we compared their contribution to adjusted R² (Adj R^2^). We first constructed a full model incorporating all fixed effects and their interactions. We then generated a set of reduced models, starting with the intercept-only model to assess baseline variance and sequentially omitting each predictor in other models. For each model, the residual variance estimates were extracted, and Adj R² was computed as: Adj R² = 1 − (Residual Variance (Reduced Model) / Residual Variance (Null Model)). The contribution of each predictor was then calculated as the difference in Adj R² between the full model and each reduced model. This approach allowed us to quantify the independent explanatory power of each predictor while accounting for shared variance in the model. We then ranked these contributions to Adj R² for each predictor.

## 3. Results

### 3.1. Cell jamming is linked to variation in cell shape across all developmental contexts

**Figure 2.**
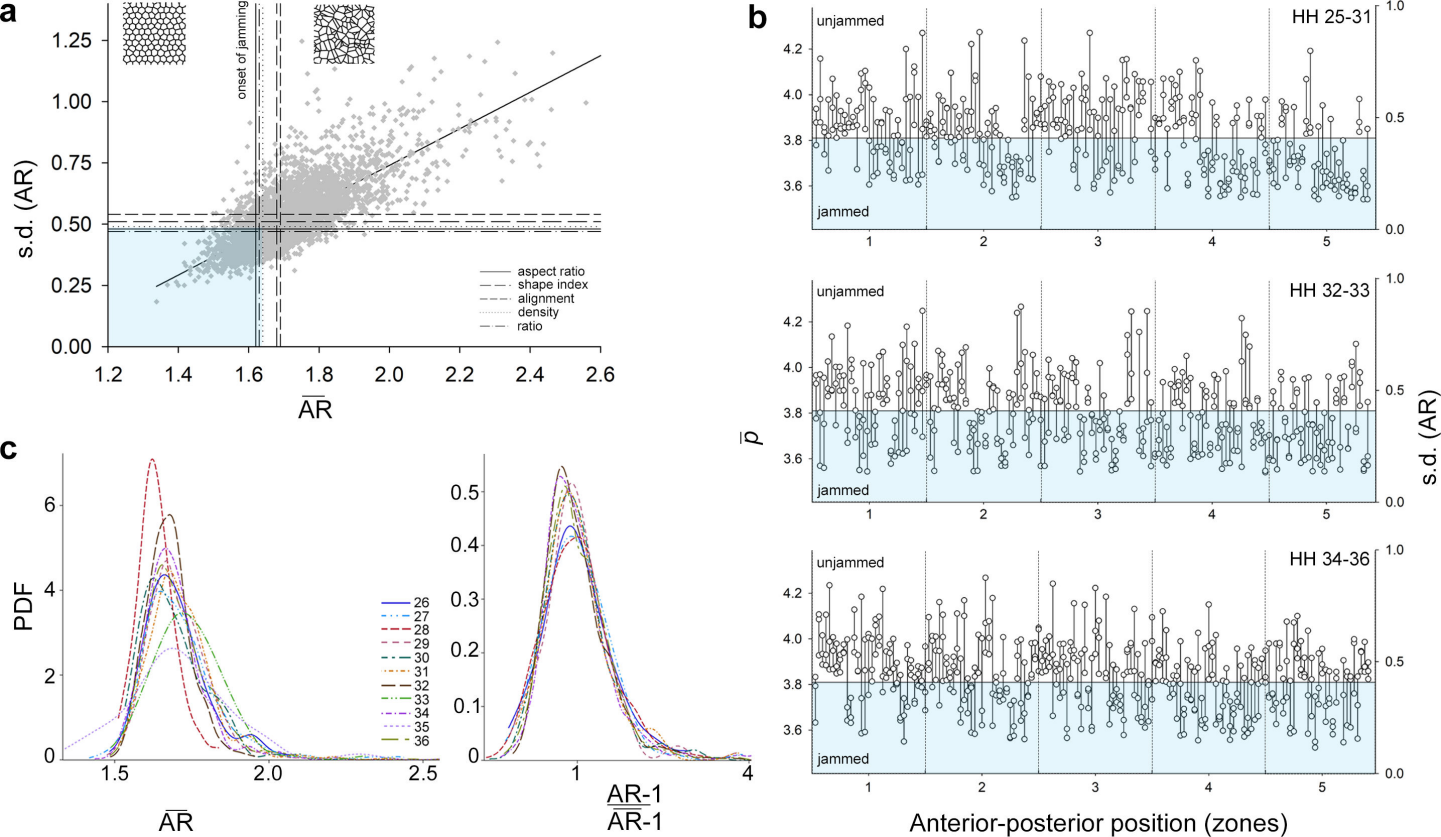
Cell jamming across biological contexts. (a) Relationship between the average cell aspect ratio ($\bar{AR}$) and its variability (standard deviation of *AR*) in each cell group, and comparison of five metrics predicting the onset of cell jamming. Shaded rectangle outlines values of ($\bar{AR}$) and s.d. (*AR*) that correspond to the agreement between all five metrics in classification of jammed state. (b) Distribution of jammed and unjammed groups of cells at the same anterior–posterior position and across developmental stages (HH 25–31 upper, HH 32–33 middle, HH 34–36 lower; electronic electronic supplementary material, table S3). Lines connect groups of cells (dots, 60–300 cells in each; a, b, c view frames in figure 1a) of the same sample. The shaded area corresponds to the onset of jamming from panel a. The left axis shows critical values of average cell shape index ($\bar{p}$) and the right axis s.d. (*AR*) for reference. (c) Probability density functions (PDF) for cell $\bar{AR}$ across developmental stages (left graph, populations combined; electronic supplementary material, figure S1b shows each population) collapse into a common distribution upon AR rescaling (right graph).

We find that cell shape (measured as aspect ratio, *AR*) and its variation (measured as s.d.) are linked: as cells in a group become more isotropic (due to more similar and equally distributed contacts with neighbouring cells), they become less variable (*b*_ST_ = 0.76, *t* = 39.66, *p* < 0.0001; figure 2a). The slope of the relationship between $\bar{AR}$ and s.d. (*AR*) did not differ across developmental stages, populations or proliferation zones (electronic supplementary material, table S2) corroborating close linkage between cell shape, its variability and tissue rigidity observed in cell monolayers and three-dimensional cell aggregations [11,56,71–73]. To examine whether packed groups of increasingly uniform and rounder cells represent a jammed state (figure 1b), we calculated the agreement between five metrics commonly deployed to predict the onset of cell jamming (see Methods). To visualize the agreement between the metrics, we show critical values associated with the onset of jamming on the plot of $\bar{AR}$ versus s.d. (*AR*) (figure 2a, $\bar{p}$* = 3.81, s.d. (α*) = 0.5, s.d. (AR*) = 0.5, density = 95%, ratio = 70%). We find that in all populations and developmental stages, groups of jammed cells are local, transient and small (having more than two jammed cell groups within a grid zone was uncommon) and randomly distributed across the grid (figure 2b, electronic supplementary material, table S3). Probability density functions (PDF) of cell $\bar{AR}$ derived for developmental stages, populations and zones are all unimodal but differ slightly in distribution (figure 2c, electronic supplementary material, S1a). We thus tested the commonality of underlying functions across these biological contexts by rescaling *AR* for each PDF to mean = 1 and min = 0. We find that all PDFs of rescaled $\bar{AR}$ collapsed into a shared density function (figure 2*c*) across widely distinct biological contexts (e.g. all developmental stages in figure 2c and all populations and zones in electronic supplementary material, figure S1b), indicating that the link between cell shape and its variation is universal across all examined contexts. Having established that the interdependence of cell shape and its variation (figure 2a,c) underlies the distribution of cell jamming across tissue span (figure 2b), we then asked whether regulatory proteins take advantage of this ubiquitous physical process.

### 3.2. Cell jamming alters spatial reach of key regulatory proteins

**Figure 3.**
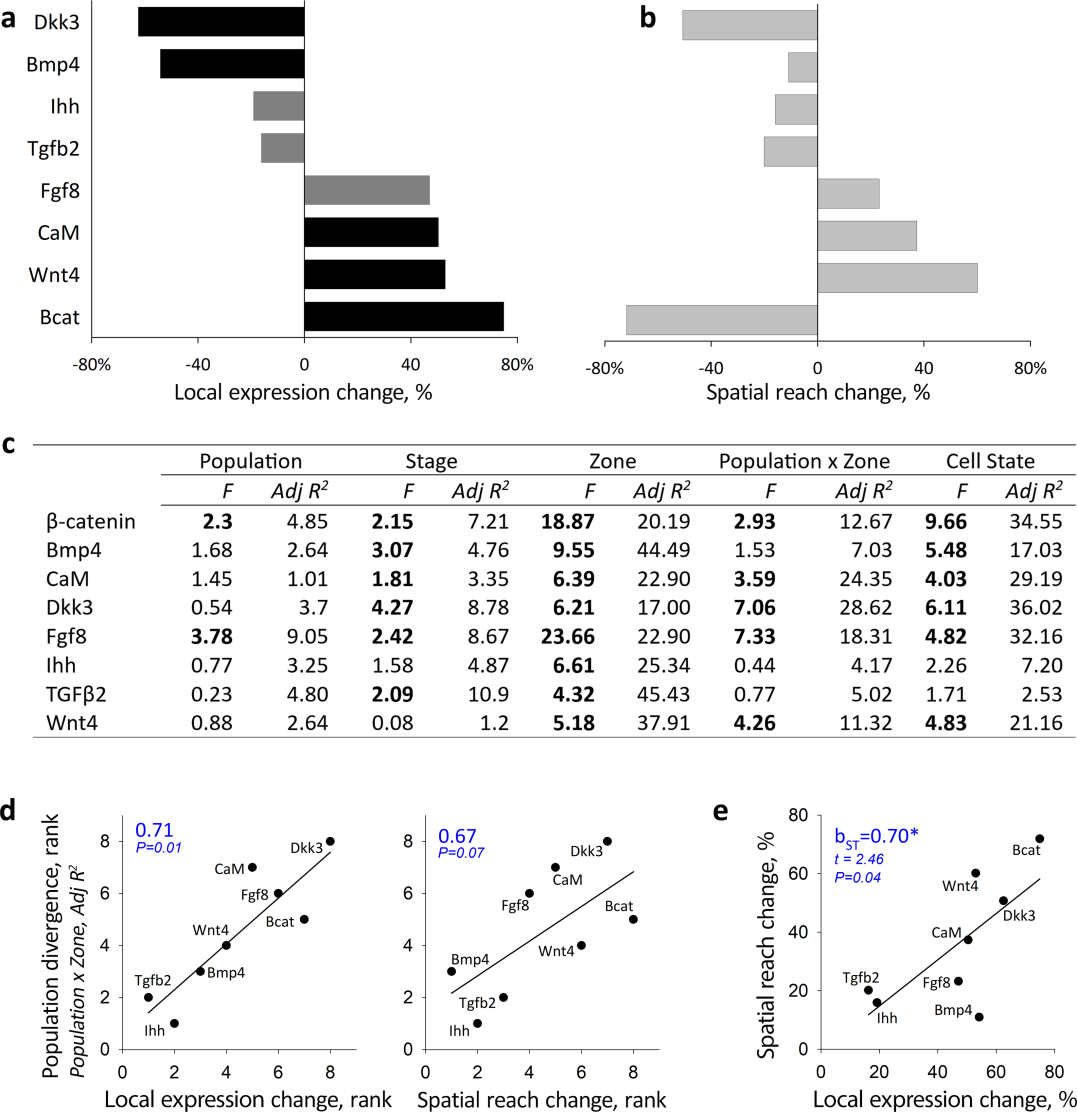
Protein expression and long-range spatial reach associated with cell jamming can prime population divergence. (a) Local (within zone) changes in protein expression between jammed and unjammed groups of cells. Bars show relative change (%) between the least-squared means (mixed effects model in (c)) for jammed and unjammed states. Black bars: *p* < 0.01, dark grey bars: *p* < 0.05 (data in electronic supplementary material, table S4). (b) Change in spatial reach of protein (a percentage of total continuous anterior–posterior length over which protein expression is correlated; data from electronic supplementary material, figure S11 and table S4) when spanning jammed versus unjammed zones. (c) Mixed effect model estimating the effects of population, developmental stage, zone, population divergence in protein gradient (Population x Zone) and cell state (jammed versus unjammed) on protein expression. Shown are *F*-values (bold shows *p* < 0.05 after within-model Sidak adjustment) and variance contribution (in %) to adjusted R^2^. (d) Cell-jamming associated change in within-zone protein expression (left) and in spatial reach of proteins (right) predict population divergence in protein gradients. Ranks are Adj R^2^ variance contributions from (c); Spearman correlation coefficients are shown. (e) Cell-jamming associated changes in within-zone protein expression predict spatial reach of the protein (absolute values, *b*_ST_ is standardized regression coefficient, electronic supplementary material, table S4).

We find that within-zone expression of Dkk3, Bmp4, Ihh and Tgfβ2 decreases when tissues are in the jammed state, whereas the expression of Fgf8, CaM, Wnt4 and β-catenin increases (figure 3a). We then examined changes in the spatial correlation of protein expression along the anterior–posterior axis of the upper beak in relation to jamming distribution (figure 3b, electronic supplementary material, S11). Following previous studies, we call this metric spatial reach (it is also called decay length, correlational span or correlational length in the literature, e.g. [22,31]). We find that under the jammed state, the expression of β-catenin, Dkk3 and Tgfβ2 becomes more restricted, whereas the expression of CaM, Fgf8 and Wnt4 becomes more spread out and uniform (figure 3b). Except for β-catenin, whose increase in local expression is associated with more fragmented global distribution (figure 3a,b), all proteins show concordant changes in the direction and magnitude of cell-jamming associated expression and its spatial reach (figure 3e). We next examine divergence in protein gradients in early beak primordia across recently established populations and the contribution of protein sensitivity to cell jamming transitions to this divergence.

### 3.3. Cell jamming can facilitate tissue-level regulatory compartmentalization and divergence

**Figure 4.**
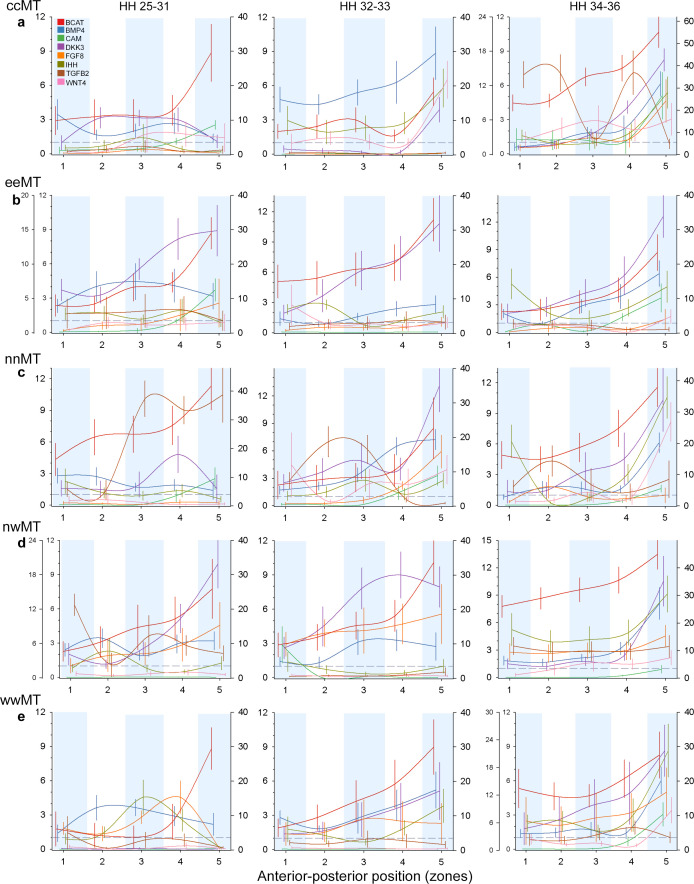
Population-specific gradients of protein expression across anterior–posterior position and developmental stages. Shown are means for each zone ± s.e. Developmental stages are arranged in columns. The left-most ordinate axis (on graphs with two left axes) shows the expression of Dkk3, and the right ordinate axis shows the expression of β-catenin. Expression of all other proteins is shown by the left ordinate axis. Grey dashed line shows the threshold of IHC detectability. Populations (electronic supplementary material, table S1): (a) ccMT—central Montana, (b) eeMT—eastern Montana, (c) nnMT—northern Montana, (d) nwMT—northwestern Montana, and (e) wwMT—western Montana.

Anterior–posterior gradients of protein expression differ among recently diverged populations (figures 3c and 4). For example, Dkk3 expression at HH 25–31 is the highest in posterior zones in eastern and northwestern Montana populations but in middle zones in central and northern Montana. Similarly, the anterior–posterior gradient of Tgfβ2 and CaM during HH 25–31 diverges among populations (figure 4). Divergence in anterior–posterior gradients is the strongest in CaM, Dkk3 and Fgf8, whereas Bmp4, Ihh and Tgfβ2 gradients are similar across populations (figure 3c). We then examine the association between protein expression, spatial reach induced by cell jamming and population divergence in protein gradients. We find that protein responsiveness to cell jamming—both in local expression and in long-range spatial reach—predicts population divergence in protein gradients (figure 3d), supporting the hypotheses that cell jamming may facilitate regulatory compartmentalization and divergence at the level of tissues (figure 1c).

## 4. Discussion

We found that transient and local cell jamming transitions, linked to the scaling of cell shape, were associated with the spatial reach of proteins across the beak in a pattern that is reflected in the regulatory divergence among populations. Close association between the activity of morphogens, transcription factors and other regulatory proteins and aspects of the cell jamming transition corroborates other studies [13,25–27,35,69] and has three main implications. First, these findings show how local transitions in fields of homogeneous cells can accomplish coordinated and region-specific cell behaviours over longer spatial scales, leading to progressive compartmentalization of tissues—a prerequisite for developmental specification and complexity (e.g. [74,75]). Second, the association between regulatory proteins’ expression and material properties of cell groups is well placed for evolutionary modifications of morphogenesis. This is because the entanglement of physical and biological processes can impose history-dependence onto otherwise ahistoric physical processes or reset contingency in biological processes. In development, such resetting allows for cycles of modification and stabilization that are necessary for differentiation and exploration, as well as for accommodation of stochastic noise [11,13,17,76]. In evolution, dynamic integration of processes with variable history-dependence allows developing systems to evolve without losing robustness [77,78] and thus transit between adaptations. Third, the effect of minimal rules that govern individual cells has long-range consequences at the level of tissues and is ultimately detectable in microevolutionary population divergence. What are the mechanisms behind these scale-transcending effects?

**Figure 5.**
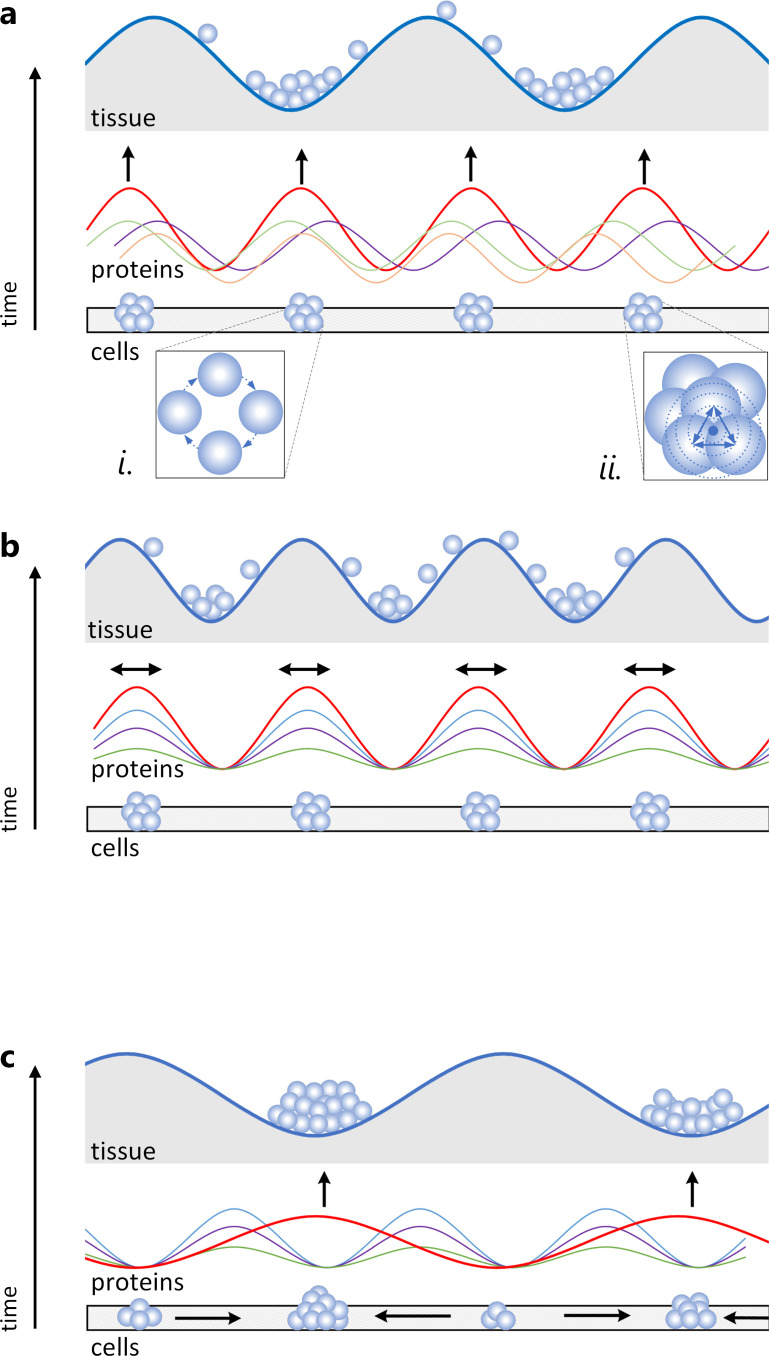
Cell jamming transitions and the origin of cell condensations. Proposed mechanisms by which transient groups of jammed cells (lower: ‘cells’ level) can be precursors of cell condensations (upper: ‘tissue’ level) that arise at the interface of the epithelium and mesenchyme. (a) Signalling (middle: ‘proteins’ level) associated with dynamic cell–cell interactions during jamming transitions prepatterns future condensations. (*i*) Jamming resets and synchronizes cell cycle variability among mesenchymal cells arising from distinct origins or arriving by different migration routes, priming them for the condensation transition, (*ii*) jamming amplifies signalling in the cell group. (b) Jammed groups form boundaries of future condensations either by juxtaposition of signalling gradients, epithelial induction, or through cell adhesion and sorting. (c) Condensations originate from jammed groups exceeding a critical size or duration of existence either through amplified signalling or through local cell adhesion.

If the transient jammed groups of NCM cells studied here are developmental precursors of ‘cell condensations’—fundamental units of cellular differentiation and morphological diversification in avian beaks [64,79–82]—then this might explain the mesoscale consequences of jamming transitions and their imprint on the patterns of population divergence (figure 3). Beak condensations arise when NCM cells, induced from the developing neural tube, migrate in distinct streams to different locations in the upper and lower beak where, upon interaction with the overlaying epithelium, they form condensations [70,83–86]. We envision three general scenarios by which transient cell jamming can be relevant to the origin of condensations (figure 5). First, jamming can synchronize cell cycles in groups (figure 5a), either by erasing differences accumulated during migration and thus priming these cells for a transition to a condensation (scenario *i* in figure 5a) or by synchronizing and amplifying signalling arising from cell–cell interactions within jammed groups (scenario *ii* in figure 5a [87,88]). Second, uniformly distributed small islands of jammed cells (figure 2b, electronic supplementary material, table S3) can create larger scale standing waves of morphogen signalling that prepattern the sites of future condensations by priming cells within these areas or affecting subsequent cell migration (figure 5b, [89]). Cell jamming transitions and associated cell-scale stresses can also delineate the boundaries of future condensations by ‘melting’ jammed tissues and channelling cell migration and accumulation or by sorting cells according to their jammed state [69,70,90–94]. Finally, condensations can originate when jammed cell groups exceed a critical size that prevents their dissociation through either mechanical or signalling mechanisms (figure 5c, [69]).

More broadly, developing systems reconcile two seemingly opposite properties—they are robust to perturbations and adaptively flexible at the same time [20,78,95]. In a systems biology view, these two requirements correspond to the properties of, respectively, an ordered phase (that enables stability and small step incremental improvements) and a disordered phase (that enables large steps in the search for novel solutions). This has led to the postulate that evolution keeps biological systems near a critical transition—just inside the ordered phase, but able to rapidly transit to a disordered phase [24,96–98]. Our finding of notable nonlinearity between the jamming state and the spatial reach of regulatory proteins, where small changes in the size and shape of individual cells can be associated with long-range protein expression, and thus expansions of developmental scale (figure 3), is characteristic of such a critical transition. These embryonic tissues, therefore, appear to hover near or repeatedly pass through critical transitions that might allow them to reconcile robust maintenance and adaptive flexibility. Under this scenario, local jamming resets or synchronizes cells, while unjamming allows large‐scale rearrangements, morphological explorations or scale expansions.

Specifically, our results corroborate the idea that components of development differ in their distance to critical transitions and that it is these dynamic changes in criticality that reconcile robustness and evolvability of development [8,23,99,100]. First, whereas the transition between levels of organization (e.g. from individual cells to tissues) might reflect critical transitions, stability is favoured within each level as is evident in the remarkably homogeneous distribution of cell shapes and jammed groups (figure 2b, electronic supplementary material, figure S1 and table S3). Second, the interchangeability of factors that lead to a jamming transition virtually guarantees that adjacent areas that vary in cell density, growth, competition, migration, adhesion or differentiation will be asynchronous in their jamming cycles as well, and thus, their distance to critical transitions. This will lead to their compartmentalization and differentiation despite uniformity of their elements. Third, some aspects of the regulatory protein network that are closely integrated with material properties of tissues (called dynamic modules in other studies) might be close to critical transitions while others remain stable [23,77,99], thereby delineating developmental variability and, ultimately, evolutionary divergence. Our finding of the variable association of protein’s spatial reach with cell jamming is consistent with this idea (figure 3, electronic supplementary material, figure S11).

The latter point raises the question of the mechanisms behind variable mechanosensitivity of protein expression in this system. In a companion study, we showed that sensitivity of transcriptional regulators to cell jamming was linked to their intrinsic disorder [62]. Specifically, intrinsically disordered proteins mediated jamming transitions through their dosage-dependent binding plasticity, acting as developmental resets, forming the dynamic modules described above and channelling developmental variation. In addition, the association of proteins’ long-range reach and cell-level jamming transitions (figure 3b) can be caused by transcellular protein trafficking or by the effect of tissue rigidity on morphogen spread [70,101].

Finally, a defining feature of criticality is expansion of the spatial reach of effects at each transition, which is also a defining feature of scaling transformations. In this respect, it is notable that the remarkable diversity of avian beak shapes can be explained by simple rescaling along a few developmental axes [33,67,68]. Developmentally, scaling transformations are often a product of modification of gradients of diffusible morphogens or growth factors, such as Bmp4 or Tgfβ2 studied here [101]. Integration of material properties of tissues with the propagation of morphogens (figure 3) enables precise control of morphogen behaviour in relation to tissue size [31,101]. Thus, local changes in cell growth parameters together with the major scale-expansion of these effects (figure 2) could well be the earliest ontogenetic origin of scaling transformations, such as those seen in avian beaks [37,102].

In sum, these results reveal rich developmental dynamics in which the cell-level changes can scale up to long-range but predictable diversifications. A combination of cell jamming with mechanosensitivity of regulatory proteins can transiently reset developmental scale, thereby priming tissue compartmentalization and enabling morphological explorations while maintaining consistency of developmental outcomes.

## Data Availability

All data are available in the supplementary materials and in data depository [45]. Supplementary material is available online.
